# Efficacy of the *Lunch is in the Bag* intervention to increase parents’ packing of healthy bag lunches for young children: a cluster-randomized trial in early care and education centers

**DOI:** 10.1186/s12966-015-0326-x

**Published:** 2016-01-08

**Authors:** Cindy Roberts-Gray, Margaret E. Briley, Nalini Ranjit, Courtney E. Byrd-Williams, Sara J. Sweitzer, Shreela V. Sharma, Maria Romo Palafox, Deanna M. Hoelscher

**Affiliations:** Third Coast R & D, Inc., 2728 Avenue Q, Galveston, TX 77550 USA; Nutritional Sciences, School of Human Ecology, The University of Texas at Austin, 1 University Station, Austin, TX 78746 USA; Health Promotion/Behavioral Sciences, Michael & Susan Dell Center for Healthy Living, University of Texas School of Public Health Austin Regional Campus, 1616 Guadalupe Street, Suite 6.300, Austin, TX 78701 USA; Epidemiology, University of Texas School of Public Health, 1200 Hermann Pressler Lane, Houston, TX 77030 USA

**Keywords:** Packed lunch, Early childhood, Parent-focused

## Abstract

**Background:**

Lunches that parents pack for their young children to eat at school or the Early Care and Education (ECE) center fall short of recommended standards. *Lunch is in the Bag* is a multi-level behavioral nutrition intervention to increase parents’ packing of fruit, vegetables, and whole grains in their children’s lunches. Designed for implementation in ECE centers, the five-week long intervention is followed three months later with a one-week booster.

**Methods:**

Efficacy of *Lunch is in the Bag* was tested in cluster randomized trial. Participants were 633 families from 30 ECE centers (15 intervention, 15 control) across Austin, San Antonio, and Houston, Texas, USA. Primary outcomes were servings of fruit, vegetables, and whole grains observed in the children’s parent-packed bag lunches. Servings of refined grains, meats/beans/eggs/nuts, dairy, chips, and sweets also were observed. Data were collected at baseline, post-intervention (6-week follow-up), pre-booster (22-weeks follow-up), and post-booster (28-week follow-up). Time-by-treatment interactions were analyzed separately for each of the food groups using multi-level models to compare changes from baseline. Analyses were adjusted for relevant demographic variables and clustering within centers and parents.

**Results:**

The intervention effected increases from baseline to 6-week follow-up in vegetables (0.17 servings, SE = 0.04, *P* < 0.001) and whole grains (0.30 servings, SE = 0.13, *P* = 0.018). The increase in whole grains was maintained through the 28-week follow-up (0.34 servings, SE = 0.13, *P* = 0.009). Fruit averaged more than 1.40 servings with no differences between groups or across time. The intervention prevented increase in sweets (-0.43 servings, SE = 0.11, *P* < .001, at the 22-week follow-up). Parents persisted, however, in packing small amounts of vegetables (averages of 0.41 to 0.52 servings) and large amounts of sweets and chips (averages of 1.75 to 1.99 servings).

**Conclusions:**

The need for and positive effects of the *Lunch is in the Bag* intervention at ECE centers where parents send bag lunch for their preschool-aged children was confirmed. An important direction for future research is discovery of more options for leveraging the partnership of ECE centers and families to help young children learn to eat and enjoy vegetables and other healthy foods in preference to less healthy choices such as chips and sweets.

**Trial registration:**

The Clinical Trials Number is NCT01292434.

## Background

*Lunch is in the Bag* is a multi-level behavioral intervention for implementation in Early Care and Education (ECE) centers to increase parents’ packing of fruit, vegetables, and whole grains in their children’s bag lunches [1, 2]. Inadequate intakes of these foods and excessive intakes of foods with added fats, sugar, and sodium are current childhood nutrition concerns in many countries [3, 4]. Healthful eating habits for young children can best be achieved by a diet that minimizes or excludes foods high in fats, sugars, and sodium, and includes a variety of dairy, meats/beans/eggs/nuts, grains/whole grains, fruits, and vegetables [4–6]. Increased vegetable intake by people older than 2 years of age has been identified in the United States as a Leading Health Indicator for the year 2020 [7]. Eating habits acquired during the youngest years persist into adolescence and young adulthood [8–10]. Children learn to like foods they are provided repeated opportunities to taste [11, 12]. Their food preferences also are influenced by being told about food and rewarded for their food choices [13–15], and they learn by identifying with their parents’ food choices and enjoyments [16–18]. Bag lunches that parents pack for their children not only provide repeated opportunities for the child to taste the packed foods but also signal the parent’s belief that the packed foods are the right ones to eat and enjoy.

Packing the child’s bag lunch to nurture healthful eating habits, however, is an opportunity rarely realized [19, 20]. Parent-provided bag lunches typically contain too few vegetables [21, 22]; seldom include whole grain items [1, 23]; contain excesses of high fat, sugar, sodium foods such as sweets and chips [24, 25]; and fail to provide appropriate amounts of important nutrients [26–28]. Parents cite family and child preferences [29] along with lack of knowledge [30, 31] and time and resources [29, 32] as barriers to providing children with recommended amounts of vegetables, whole grains, and other healthy choices. When parents were asked in group interviews what can be done to help them pack better lunches for their preschool children, they expressed interest in receiving nutrition information from their children’s ECE centers, indicated desire for regular feedback about what their children eat, and recommended recipe exchanges and other methods that facilitate parents talking to and learning from each other [33]. *Lunch is in the Bag* was developed to address these parent-identified levers and barriers to packing healthy bag lunch for preschool aged children. Implemented in ECE centers where parents are required to send bag lunch for their children, the five-week long intervention was followed three months later with a one-week booster.

The primary hypothesis of *Lunch is in the Bag*’s cluster randomized control trial was that children’s lunches in the intervention group would, on average, contain more servings of: a) fruit, b) vegetables, and c) whole grains at the 6-week and 28-week follow-up periods compared to children’s lunches in the control group. The study design also enabled examination of the hypothesis that increasing fruit, vegetables, and whole grains in the parent-packed bag lunch would displace chips and sweets thereby increasing children’s exposure to a healthy meal pattern in the lunch they bring from home.

## Methods

### Study design

**Fig. 1:**
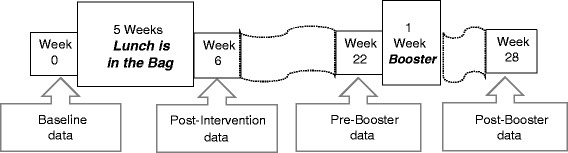
Data collection schedule. Packed lunches were observed four times at intervention and at control ECE centers

A total of 30 ECE centers where parents supply bag lunch for their child were recruited in central and south Texas with 15 centers randomized to the *Lunch is in the Bag* intervention and 15 centers to a wait-list control condition. As is depicted in Fig. 1, data were collected pre-intervention (baseline), post-intervention (6-week follow-up), pre-booster (22-weeks follow-up), and post-booster (28-week follow-up). The four measurement periods enabled analyses of initial behavior change achieved with five weeks of intervention in the fall of the school year, maintenance of the behavior change after removal of the weekly messages and activities, and “boosting” or recovery or other response to reintroduction during a single week of *Lunch is in the Bag* in the spring of the school year.

### Intervention

**Fig. 2:**
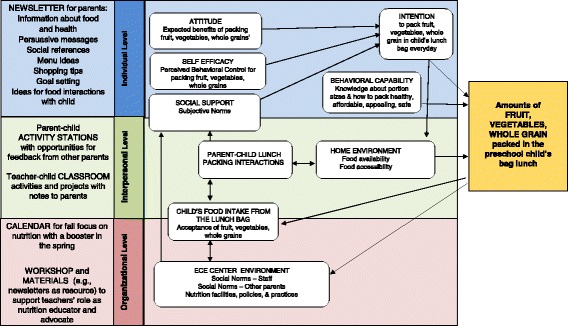
*Lunch is in the Bag* intervention logic model

Grounded in Bandura’s Social Cognitive Theory [34], the social-emotional-cognitive Theory of Reasoned Action [35], and an ecological approach that focuses on people and environments as targets for health promotion [36], the intervention included 16 of the 26 behavior change techniques identified in the taxonomy of techniques used in interventions to increase healthy eating (e.g., provision of information, prompting intention formation, providing feedback on performance, using follow-up prompts, provision of opportunities for social comparison) [37]. The intervention goal was parents’ packing one serving each of fruit, vegetables, and whole grain items in their preschool children’s bag lunches. Intervention components at individual-, interpersonal-, and organizational-levels were designed using Intervention Mapping [38] and developed with formative feedback from representatives of the population of intended implementers and stakeholders. *Lunch is in the Bag*’s logic model linking the intervention strategies and techniques to the theory-grounded behavioral and environmental targets for change is depicted in Fig. 2.

At the individual-level, the core component was handouts/newsletters sent to the parents from the ECE center to provide information to develop or reinforce knowledge about portion sizes and how to pack lunches that are healthy, safe, and appealing for preschool-aged children. Each issue of the newsletters provided sample menus with shopping and food preparation tips for saving time and money, presented images and social journaling references to promote and reinforce positive attitudes and sense of self-efficacy for packing affordable healthy lunch, challenged parents to “try something new” thereby prompting goal setting and intention formation, and encouraged parent-child interactions to increase variety in the diet and enjoy nutrition fun at home.

Components at the inter-personal level were designed to build social support for parents’ packing of fruit, vegetables, and whole grains in their children’s lunches every day. *Lunch is in the Bag*’s lesson plans for the children’s classrooms included activities (e.g., using a colorful placemat to help the children recognize the “My Plate” food groups of the items packed in their lunch bags) and projects (e.g., building a whole-grain train on the classroom walls) to encourage the children to request, eat, and enjoy vegetables and whole grain foods and other healthy items in their lunches. The classroom kit also provided notes for teachers to provide performance feedback to parents (e.g., sending home a “gold medal” certificate when the child’s lunch included foods from all five of the “My Plate” food groups) and to prompt the parents to engage with their children at home in talking about and supplying food (e.g., “Please remember to send a favorite fruit for Favorite Fruit Friday”), materials (e.g., empty food packages of whole grain cereal for the grain train), and family participation (e.g., sending a favorite vegetable recipe for family recipe exchange) that were needed for the classroom activities and projects. Cues to action and other messages from the newsletters and classroom lessons were reinforced in parent-child activity stations placed in a common area of the ECE center to provide hands-on games and question-and-answer interactives for the parent and child to complete together. The activity stations also provided opportunities for social comparison via parent-to-parent sharing (e.g., “Tell us about your Gold Medal Kids! On the sticky notes provided, write down a positive change your child or family has made and post it here”).

At the organizational level, an implementation support package was developed to include the *Lunch is in the Bag* center- and classroom-level implementation calendars for five weeks of focus on nutrition in the fall of the school year followed-up with a booster in the spring approximately 22 weeks after start-up. The classroom-level kits included extra copies of the newsletters for teachers to use as a resource throughout the school year, the classroom lesson plans and materials. The center-level kit included the newsletters to send to parents and materials for the parent-child stations. *Lunch is in the Bag* Weekly Activity Logs for the Center Director and for the classroom teachers were augmented with a one hour workshop to orient ECE staff to the intervention components and to support them in their role as nutrition educators and advocates.

Pilot studies of *Lunch is in the Bag* showed that it increased the amount of vegetables and whole grains that participating parents packed in their children’s bag lunches [1, 39, 40]. But, despite the positive changes, the estimated percent of children whose mid-day meal provided daily exposure to a serving of the target foods remained less than 20 % for vegetables, less than 25 % for whole grains, and near zero for daily exposure to a serving each of vegetables, fruit, and whole grains [23]. The data- and theory-driven adjustments that were made to strengthen the original intervention included creating the classroom activity in which the child earned a "gold medal" certificate to take home to the parent(s) when the lunch contained items from all five of the "My Plate" food groups (dairy, meats/beans/eggs/nuts, vegetables, fruit, grains) [5], branding the intervention materials with a gleeful squirrel character to remind that fruit and vegetables and whole grains are fun to eat, and adding the "booster" week three months after the original five-week intervention.

Content and formatting were developed in consultation with an Advisory Group constituted of parents, ECE teachers and directors, and nutrition and behavioral science experts. Formative work included interviews with parents and ECE teachers and directors to evaluate and improve clarity and appropriateness of the messages and images. All narrative materials were produced at a sixth grade reading level to accommodate a wide range of education levels of parents and teachers of young children. An overview of the topics and activities shown by component and week of intervention is presented in Table 1.

**Table 1:** Topics shown by component and week of the *Lunch is in the Bag* intervention

Week	Newsletter sent from the Center to the Parent	Parent-Child Activity Station	Teacher-Child Classroom Activities	Teacher-Parent Notes for Classroom
1	Lifelong eating habits!	Match food pictures to MyPlate food group colors	MyPlate Placemat to use every day and support the Lunch Colors activity	Please send a favorite fruit for Friday snack
	--MyPlate food groups			
	--Serving sizes			
	--Nutrition health facts			
2	Read the Label First!	Match whole grains to their pictures	Grain Train constructed around the classroom from empty boxes	Please send empty packages for Grain Train AND whole grain item for Thursday snack
	--Nutrition facts labels			
	--Whole grain sources			
	--Sugar sources			
3	Make sure it’s safe!	Match fruit to their colors	Wash Those Germs (glitter)	Please send favorite vegetable for Wednesday snack
	--Packing food safely			
	--Keeping food & child safe			
4	Make it appealing!	Match vegetables to their colors	Favorite Family Meal with all 5 food groups drawn on a paper plate	Please send favorite vegetable recipe to share
	--Making lunch fun			
	--Lunch packing tips			
	--Shopping hints			
5	Beyond the bag!	Match the food to Gold Medal Lunch	Mystery Fruits & Vegetables in bags for the children to touch, smell, & tell	Please send favorite vegetable recipe AND a vegetable for Wednesday snack
	--Vitamins & minerals			
	--Introducing new foods			
	--Cooking fun and easy			
6	Lifelong eating habits!	Match food to Gold Medal Lunch	Who can tell "What's Missing from My Plate?"	Please send vegetable for Wednesday AND whole grain for Thursday snack
	--Serving sizes			
	--Healthy eating hotline			
	--Nutrition facts label			
All 6	--Menu suggestions	Information sheets to take away	--Book at circle time	Gold Medal certificate when Lunch Colors shows all 5 food groups
	--Try something new		--Lunch Colors	
	--Nutrition fun at home		--Tracking Fruit & Veg	

### Setting

ECE centers were eligible to participate if they were licensed by the Texas Department of Family and Protective Services to provide ECE and had enrollment of at least 15 children aged 3 to 5 who regularly ate parent-provided bag lunch at the center. At intervention centers, *Lunch is in the Bag* was made available to all families whose children were in classrooms with ages 3-5 years irrespective of whether or not any given family enrolled in the study. At control ECE centers, *Lunch is in the Bag* was made available at the beginning of the school year following the completion of their participation in the trial.

### Study population

Families were recruited into the study as parent-child dyads in which the "parent" was the adult family member primarily responsible for packing the child's lunch and the "child" was aged 3-5 years and regularly ate bag lunch at the ECE center. Enrollment in the study was limited to one parent-child dyad per family.

The ECE directors and the lead teachers of classrooms that included children ages 3-5 years were recruited to participate in documenting context and evaluating implementation of *Lunch is in the Bag* at the ECE center.

Informed consent procedures and research protocol were approved by the Institutional Review Boards of the University of Texas Health Science Center in Houston and The University of Texas at Austin. The recruitment and consent documents were prepared in English and in Spanish language and arrangements were made to provide translators or to produce the newsletters in Spanish language if needed or requested.

### Data collection

A structured food record was used to document direct observation of the contents of the children’s lunch bags at intervention and control centers on two randomly selected non-consecutive week days at each of the four measurement periods. Content of the bag lunches was observed in a room separate from the children approximately 20 to 60 min prior to the children’s lunch time. When a child was absent on lunch observation days, arrangements were made to return as soon as possible to observe that child’s lunch. Data collectors were not blind to any center’s assignment to intervention versus control because data were collected on-site at the ECE centers and the intervention included materials that were posted in common areas of the center.

Food observers were trained with a two-day research-based protocol [41]. The training included pre-test with 10 sample lunches, stations for the observers to create food items in order to learn what to look for (e.g., making sandwiches with different kinds of bread to learn to distinguish whole grain bread), and post-testing with 10 sample lunches. Retraining was required for observers who failed to correctly identify 90 % of the items/ingredients and achieve 80 % agreement to actual measured portion for 90 % of the food and beverage items.

The training method was tested by comparing intra-class correlation coefficients (ICC) and Pearson’s correlation coefficients calculated for the ratings of portion sizes by each of the first group of 11 trained observers. The Pearson correlation coefficients were very close to the ICCs (to the second decimal place). Overall average was based on the ICCs, and was adjusted using the Spearman-Brown correction. ICCs were averaged across observers to obtain an inter-rater reliability coefficient (IRCC). The obtained IRCC was 0.979 [41].

In the field, a separate food record was completed for each lunch by the single observer assigned to that lunch bag. The observer recorded a nominal description of each food item, specified the observed amount in standard measuring units (such as 1/2 cup of carrot or 1 slice of bread or 4 Tablespoons of raisins), and post-coded each item’s food groups and the number of age-appropriate servings based on guidelines published by the U.S. Department of Agriculture’s Child and Adult Care Food Program (CACFP) [42]. The food groups coded were fruit, vegetable, whole grain, refined grain, dairy, protein (meat/beans/eggs/nuts), sweets, and chips. Sweets as a coding category was comprised of sugar-sweetened-beverages and sugary confections such as candy and cookies. Chips as a coding category was comprised of salty snacks that are high in added fats and sodium such as potato chips and cheese crackers.

To ensure that minimal measurement drift was introduced during the extended study period, the registered dietitians who provided the data collection training validated 10 % of each trained observer’s recorded lunches during each observation period and provided coaching and refresher training when needed. A final step in the food observation protocol was having a registered dietitian who was one of the trained observers review and verify the accurate coding of food group and serving size for every item from every food record.

Data from the verified food records were used to measure parents’ lunch packing behavior. The primary measure was number of age appropriate servings of fruit, vegetables, and whole grains parents packed in the children’s lunches. The numbers of age appropriate servings of refined grains, meats/beans/eggs/nuts, dairy, chips, and sweets also were analyzed. In addition, occurrences of the foods were analyzed because increased numbers of servings could result from increased portions of the food but also could result from increased prevalence of parents packing the food at least occasionally and/or increased habit of packing the food in the child’s bag lunch [23]. Prevalence of parents who packed the targeted food was measured as percent of parents packing the food on at least one of the two days in the measurement period. Parents’ habit of packing was measured as percent of lunches containing the specified food. Packing lunches that present healthy meal patterns was measured as percent of lunches containing foods from all five of the “My Plate” food groups and percent of lunches containing zero sweets and chips.

*Contextual* data were collected at baseline. A questionnaire for the ECE center director asked about characteristics of the center. Questionnaires for the teachers asked for self-report of demographic information. Questionnaires for the parents asked for self-report of demographic information including asking the parent to self-report height and weight. The parents also completed a demographic questionnaire for the child, but the child’s height and weight were measured by the trained data collectors following standard protocols for height and weight measurement previously used in nutrition research with school children [43]. Digital platform scales with remote display (Tanita Professional, BWB-800S) and portable stadiometers (Perspectives Enterprises) were used. The equipment was calibrated by the trained data collectors before measurement at each center. Inter-rater reliability (ICC = 0.998) for the standard protocol was determined in prior work [43]. Body Mass Index (BMI) was calculated using the standard method and values compared to the U.S. Centers for Disease Prevention and Control (CDC) standards for weight status. BMI > 85^th^ and < 95^th^ percentile was considered overweight and BMI > 95^th^ percentile was considered obese [44].

*Implementation* data were collected using mixed methods and coded using criterion references and MultiAttribute Evaluation (MAE) methods [45, 46] to assess amounts and qualities of use of the intervention and its usefulness and usability from the perspectives of the parents and the staff at the intervention ECE centers. In accord with recommendations for theory-driven process evaluation [47], preparation for *Lunch is in the Bag*’s efficacy trial included the mapping of an action model of implementers' use of the intervention components. The action model guided development of the process evaluation tools.

The Activity Logs completed weekly by the ECE directors documented distribution of the newsletters to parents and installation and maintenance of the parent-child activity stations and also provided check-list feedback about the director’s monitoring and support of the classroom activities. The classroom teachers’ weekly Activity Logs documented which classroom activities were implemented, distribution of Gold Medal certificates to parents when the child’s lunch included all five food groups, and the rate of parent response to the notes requesting items to support the classroom activities.

Summary evaluation questionnaires were completed by parents and ECE staff at the conclusion of the fifth week of *Lunch is in the Bag* to record their experience of the intervention. The questionnaires asked how many and how much of the newsletters they had read, their recall of the content of the newsletters and the stations, and their evaluation of the intervention. The questionnaire for parents also asked for report on an ordinal scale (0 = not at all, 1 = some, 2 = a lot) of “how much your child talked to you” about each of 12 different classroom activities.

Direct observation of implementing actions and artifacts was conducted by the same data collectors who were trained to observe the contents of the children’s parent-packed bag lunches. They participated in a half-hour training and received a copy of the “self-training script” on how to use Innovation Configuration (IC) tools per the Concerns Based Adoption Model (https://www.sedl.org/cbam/innovation_configurations.html) to measure amounts and qualities of implementation observed on one randomly selected half-day at each of the intervention centers. Separate IC tools were developed for observing the parent-child learning station, the classroom circle time and snack time activities, and the lunch time teaching-learning activities. Each item on each tool was displayed with an accompanying item-specific rubric that described the quality of implementation that represented 0=”not done” to 5=”the expert way of doing.” The tool for parent-child activity stations assessed placement, visibility (e.g., 5=”well lit, clearly visible”), accessibility, completeness, cleanliness, and also provided the observer the opportunity to comment on apparent use of the station. The tool for the lunch-time teaching-learning activity assessed proportion of the children with which the teacher interacted during the “lunch colors” food group identification activity, proportion of gold medal certificates that were appropriately awarded, and qualities of interacting with the children whose lunches were ineligible for gold medal. The tool also provided rubrics for assessing five dimensions of food knowledge development (e.g., using classroom books and materials to highlight the variety of fruits and vegetables in the children’s lunches).

The Director questionnaire that was administered to measure context at baseline also was administered at the 28-week follow-up and used as a data source for measuring nutrition advocacy via center-level policies and practices. The items described whether nutrition education was integrated into the center’s curriculum for ages 3 to 5, characteristics of the nutrition education provided to the children (e.g., how often nutrition lessons are taught), the type and frequency of nutrition education provided to the children’s parents, and a set of questions asking the director’s opinion about the importance of nutrition education for children and their families, and the frequency and importance of teachers talking with children and with the children’s parents about healthiness of the children’s eating habits.

Items from the process evaluation tools were used as single, equal weight, additive “location measures” at the roots of multi-attribute evaluation [MAE] trees [45] whose branches and nodes were specified by the process evaluation team members to represent three domains of implementation: (1) use of the intervention components, (2) user experience of the intervention’s usability, and (3) user experience of the intervention’s usefulness. The obtained raw score for each item was recoded into a “location” on the given item’s raw score scale after the evaluation team set the lower bound (i.e., 0 % of the value range) at the “worst” or least desired answer and the upper bound (i.e., 100 % of value range) at the “best” or most desired answer. Items with yes/no response options (e.g., teachers’ Activity Logs asked for a check mark for each of the classroom activities for the given week) were scored 0 or 100, and items for which there were ordinal response options were assigned numerical anchors that represented the team’s consensus about “location” of the given response on the 0 to 100 scale (e.g., teachers’ Activity Logs asked for effectiveness ratings for each classroom activity for each week on a scale labeled excellent, good, fair, poor and the team set the scoring as excellent = 100, good = 75, fair = 50, poor = 25, and not completed = 0).

Data were aggregated by assuming equal weights at each level of branching in the MAE trees. For example, aggregating the location measures to score the branch representing the extent to which any given teacher did all of the classroom lessons with the children began by assigning 0 or 100 based on whether or not the teacher marked “completed” for the given type of activity. The second step was to weight the result by multiplying it by 0.167 (because there were six weeks of activities) and then adding it to the weighted result for the other five weeks. The third step was to weight the aggregated total across weeks for the given type of activity by 0.25 (because there were four types of classroom activity), and then aggregate by adding to the weighted results for the other three types of classroom activity (the four types of activity are shown in Table 1: lunch time activity, circle time book reading, snack activity, and an activity to engage the children in making and using an artifact such as the grain train). The result for the tree branch representing “teachers did the classroom lessons with the children” was then multiplied by 0.20 and added to the weighted result for the other four branches listed in Table 2 on the node representing use of *Lunch is in the Bag*’s classroom component. Three of those branches had 0, 100 scores at the level of the location measures, but scoring of the branch representing “classroom activities were configured as intended” was based on the single half-day of direct observation recorded on the Innovation Configuration tools by the study team’s trained observers. The numerical raw scale scores anchored to the narratives on the rubrics presented on the Innovation Configuration tools were assigned values”not done” = 0, 1 = 20, 2 = 40, 3 = 60, 4 = 80, and 100=”expertly done” and then weighted by the number of items for the given classroom activity (e.g., there were five items with accompanying rubrics for evaluating the classroom lunch time activity, and the aggregate score for that activity was, therefore, obtained by multiplying each item’s assigned value by 0.20 and then adding across the items).

**Table 2:** Numbers of intervention centers (*N* = 15) shown by scores indexing each center’s implementation of *Lunch is in the Bag*

MultiAttribute Evaluation (MAE) tree with • branches and ○ nodes that enabled aggregation of location measures from the process evaluation tools across attributes and components of implementation in the □ domain representing use of *Lunch is in the Bag*	Low (score 0–50)	Medium (score 51–75)	High (score 76–100)
• ECE center staff sent parents the newsletters^1^	0	0	15
• Parents recalled receiving the newsletters^2^	0	2	13
• Parents read the newsletters^2^	0	2	13
• Parents recalled newsletter content re: fruit, veg, whole grain^2^	0	1	14
• Parents recalled newsletter content re: home practice activities^2^	0	6	10
• ECE center staff recalled content of the newsletters^3^	3	2	9
○ Newsletters sent, read, recalled	0	2	13
• ECE center staff installed the parent–child activity stations^1^	0	1	14
• Parent–child activity stations were configured as intended^4^	6	0	9
• ECE center staff recalled content of the parent–child stations^1^	2	0	13
• ECE center staff saw parents visit the parent–child stations^1^	8	6	1
• Parents recalled content of the parent–child activity stations^2^	2	11	2
○ Parent–child activity stations installed, visited, recalled	2	8	5
• Teachers did the classroom lessons with the children^1^	0	4	12
• Classroom activities were configured as intended^4^	9	6	0
• Teachers sent classroom notes to parents^5^	0	5	10
• Parents recalled classroom notes from the teachers^5^	0	10	5
• Parents recalled child talking about the classroom activities^2^	15	0	0
○ Classroom activities done, supported, talked about	0	15	0
• ECE center had child nutrition education in its curriculum^6^	5	3	7
• ECE center had policies re: nutrition education for parents^6^	7	8	0
• ECE center had informal policies/leaders support for nutrition^6^	4	7	4
○ Nutrition education and behavior advocated at the ECE center	7	7	1
□ Use of the intervention	3	10	2
Other items from data sources 2 and 3 were input to score multi-attribute evaluation (MAE) trees for:			
□ Usability of the intervention from the users’ perspectives	0	9	6
□ Usefulness of the intervention from the users’ perspectives	0	3	12

Data sources of the location measures at the bottom of the MAE trees: 1 = ECE Directors’ Weekly Activity Logs, 2 = Parent Summary Evaluation, 3 = ECE Staff Summary Evaluation, 4 = Innovation Configuration Observation Records , 5 = ECE Teachers’ Weekly Classroom Activity Logs, 6 = ECE Director Questionnaire

The MAE strategy for coding location measures as “percent of range” on each item’s own raw score scale enabled aggregation across different types of data obtained with different methods of collecting data from different numbers of people to provide quantitative summary statements at the branch and node levels (e.g., data from the Center Director’s Activity Log were combined with survey data obtained from multiple parents and multiple teachers to score use of the newsletter component of *Lunch is in the Bag*) and then aggregate across the nodes to produce domain scores for implementation as a multi-dimensional construct.

### Statistical analyses

The input for analyzing outcomes of implementing the *Lunch is in the Bag* intervention was the measures of parent lunch packing behavior. Separate multi-level models were employed to analyze outcomes for each of the food groups. Each model compared the follow-up with the baseline measure. Parental lunch packing behaviors can cluster within centers and within parents, both across the two lunch observations obtained within the measurement period and across measurement periods. Accordingly, three-level regression models were constructed to allow random effects at the center level, as well as at the parent level, within and across time periods. In addition, models were adjusted for the child’s gender and age and for parent BMI, ethnicity, marital status, and education as these variables were identified in preliminary analyses as potential confounders. Servings and occurrences of the foods were estimated at each measurement period and treatment condition. Intervention effects were evaluated with a time-by-treatment interaction term. Analyses of intervention outcomes were conducted using Proc Mixed or Proc Glimmix, depending on the distribution of the outcome, in SAS (version 9.2, SAS Institute, Inc., Cary, NC, USA) for restricted maximum likelihood (REML) estimation.

## Results

### Center characteristics

Size of the ECE centers ranged from 30 to 300 children (median = 90). At 13 of the centers 3 year olds and 4 year olds were in separate classrooms, but at 4 centers 3 year old children shared classrooms with 2 year olds, and at 12 centers they shared classrooms with 4 year olds.

Twenty of the ECE centers were affiliated with a church or synagogue, 5 were affiliated with local or nationwide organizations or chains, and 5 were stand-alone organizations. All 30 of the ECE centers were retained in the study through the 28-week follow-up.

**Fig. 3:**
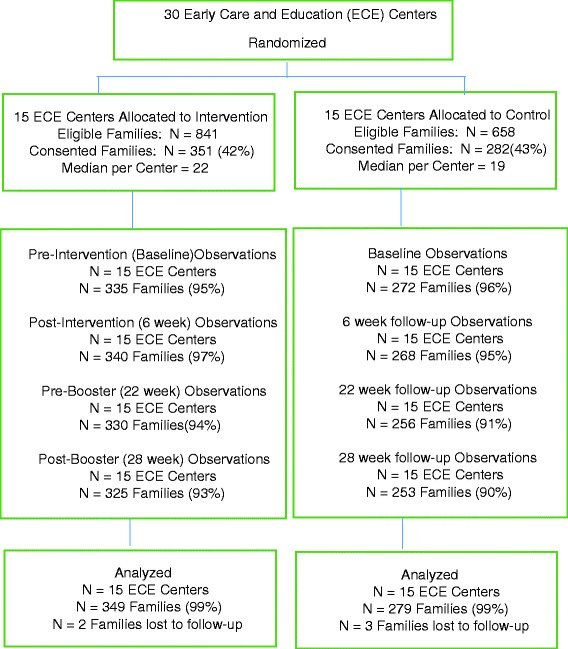
Study flow

With 42 % of eligible parent-child dyads consenting to participate, the total number of families enrolled in the study was 633. Nearly all of the consented families (91 %) were retained in the study through the 28-week follow-up. Numbers enrolled per center ranged from 12 to 43 (median = 22) in the intervention group and from 6 to 30 (median = 19) in the control group. The differences in sizes and study enrollment rates of the ECE centers resulted in more study participants at the intervention centers (*n* = 351 parent-child dyads) than at the control centers (*n* = 282 parent-child dyads). The numbers of parent-child dyads for whom lunch bag observations were available at each of the measurement periods (607 at baseline, 608 at 6-week follow-up, 586 at 22-week follow-up, and 578 at 28-week follow-up) are shown in Fig. 3.

### Family demographics

Differences in sizes and constituencies of the ECE centers resulted in demographic differences in the intervention group compared to the control group. The intervention group had relatively larger proportion of Hispanic children and parents, fewer parents older than 34, fewer parents with overweight or obesity, more parents with college degree, fewer single parents, and higher annual family income. Statistical analyses of the outcomes data included adjustment for these demographic differences, with education preferred over income as a covariate because it had fewer missing values.

Across intervention and control groups, the "parent" member of the parent-child dyad in the majority of the participating families had BMI indicating healthy weight (61 %), was of non-Hispanic White heritage (72 %), possessed a college or advanced degree (82 %), and had annual family income greater than $100,000 (57 %). Nearly all (90 %) of the “parent” members of the parent-child dyads were female and married or partnered. Average age was 36. Half (50 %) of the “child” members of the participating parent-child dyads were ages 2 to 3 and half were ages 4 to 6; 52 % were male; 22 % had BMI indicating overweight or obese, and 6 % were underweight. Although a few of the parents (*N* = 36) reported the primary language in the child’s home was other than English (Spanish = 20; Hebrew, Vietnamese, Urdu, or other Asian language = 14, European or African language = 2), none of the parents indicated need or request for translations of the study consent forms or intervention materials.

Parents’ answers at baseline to a questionnaire measuring their psycho-social dispositions toward packing fruit, vegetables, and whole grains in their children’s bag lunches showed that the majority (46-73 %) lacked knowledge about age-appropriate portion sizes of these foods for their 3 to 5 year old child and were more favorably inclined toward packing fruit and whole grains than toward packing vegetables. Proportions of parents indicating that they pack the target food because “my child likes them,” for example, was 50 % for vegetables compared to 79 % for whole grains and 92 % for fruit; “ease of packing” as a reason for packing the foods was 91 % for fruit and 88 % for whole grains compared to 74 % for vegetables; and prevalence of intentions to pack the foods every day for the next three weeks was 92 % for fruit and 75 % for whole grain foods but only 51 % for vegetables. The percent of parents who reported they pack the foods because “they are inexpensive,” however, was comparable across the three foods: 65 % for fruit and 68 % for whole grains and for vegetables.

### Teacher demographics

The majority of the teachers were non-Hispanic White (55 %) and had worked in the ECE field for 8 years or longer (54 %). Nearly all were under 55 years of age (46.5 % aged 35-54 years and 38 % aged 18-34 years). More than 75 % reported some type of formal nutrition education through in-service or other training or coursework.

### Implementation of *Lunch is in the Bag*

Results of the MAE analyses for the 15 ECE centers that were in the intervention group are presented in Table 2 and were based on input from 90 ECE teachers and directors, 244 parents, and the single site visits made by single members of the study team to each center to record his or her direct observations of implementing actions and artifacts. Newsletters for parents was the component that most consistently obtained high implementation scores (i.e., at the aggregate level for this component, 13 of the 15 intervention centers obtained a high score). In contrast, only one center obtained an implementation score in the high range (i.e., 76 to 100) for center-level advocacy of child and family nutrition education and behavior. Only 5 of the 15 centers obtained high scores for implementation of the parent-child stations. All 15 obtained a medium score for implementation of the classroom component. Aggregate analyses at the domain level of the MAE trees showed that the users/implementers (i.e., ECE directors, teachers, and parents) experienced the intervention as useful (high scores at 12 of the 15 intervention centers) and as usable for some ECE centers and families (high scores at 6 of 15 centers), but that the intervention was used as fully as the developers intended at only 2 of the 15 centers.

Responses to items from the teachers’ Activity Logs and the summary evaluation questionnaires completed by the ECE center staff and parents at the conclusion of the fifth week of *Lunch is in the Bag* also were investigated in separate analyses to provide a cross-site summary of implementation. These analyses showed 71 % of the teachers reported they did all of the classroom activities; teachers reported that approximately half (54 %) of parents sent the items requested for classroom activities; 83 % of parents reported that they had read the newsletters, 60 % indicated they had visited the center-based activity stations, but only 4 % recalled hearing their children talk about the classroom activities.

### Common foods in the children’s lunches

Item level tallies of foods observed in the children’s bag lunches at baseline showed the top 15 in descending order were (1) sweetened fruit drink, (2) grapes, (3) refined grain breads, (4) apple sauce, (5) 100 % whole wheat bread, (6) strawberries, (7) cheese crackers, (8) apple, (9) carrots, (10) snack crackers other than cheese crackers, (11) refined grain pasta, (12) fruit leather or similar sweetened fruit snacks, (13) 100 % fruit juice, (14) cookies, and (15) tortilla chips or corn chips. The percent of lunches containing any amount of the coded food groups were 74 % containing fruit, 48 % containing sweets, 30 % containing vegetables, and 22 % containing chips. Whole grains were packed in 17 % of the lunches with 10 % exclusively whole grain and the other 7 % a mix of whole and refined grain items.

### Changes in servings of the targeted foods parents packed in their children’s bag lunches

Information about numbers of age-appropriate servings of fruits, vegetables, and whole grains observed in the total of 4712 parent-packed bag lunches is presented in Table 3. The amount of fruit averaged approximately one and half servings with no differences between groups or across time. At the intervention centers the amount of vegetables increased from less than half a serving (0.37) to slightly more than half a serving (0.52) at the post-intervention 6-week follow-up, whereas the amount of vegetables in the children's lunches at control centers was less than half a serving (ranging from 0.24 to 0.29 servings at the different measurement points) and did not change from baseline. The change in servings of vegetables parents packed in their children’s lunches was, therefore, significantly larger at intervention than at control centers (difference of 0.17 servings, Standard Error (SE) = 0.04, *P* < 0.001). The intervention’s effect on amount of vegetables packed degraded during the three months after the intervention. At the pre-booster 22-week follow-up, parents at intervention centers packed less than half a serving (0.41 servings) and the amount was not significantly different from baseline. At the post-booster 28-week follow-up, the amount of vegetables parents at intervention centers packed in their children’s lunches was still less than half a serving (0.45 servings) but was significantly increased relative to baseline (*P* = 0.009) although not significant in the treatment-by-time analysis. At none of the measurement periods did the parents’ packing of vegetables attain *Lunch is in the Bag*’s target of a full serving.

**Table 3:** Numbers of servings of foods from the “My Plate” groups observed in the children's bag lunches

	Intervention Mean (Standard Error)	Control Mean (Standard Error)
Fruit		
Pre-Intervention Baseline	1.56 (0.12)	1.46 (0.12)
Post-Intervention 6-week follow-up	1.61 (0.12)	1.44 (0.12)
Pre-Booster 22-week follow-up	1.56 (0.12)	1.41 (0.12)
Post-Booster 28-week follow-up	1.64 (0.12)	1.49 (0.12)
Vegetables		
Pre-Intervention Baseline	0.37 (0.05)	0.27 (0.05)
Post-Intervention 6-week follow-up	0.52 (0.05)***,******	0.24 (0.05)
Pre-Booster 22-week follow-up	0.41 (0.05)	0.27 (0.05)
Post-Booster 28-week follow-up	0.45 (0.05)**	0.29 (0.05)
Whole grains		
Pre-Intervention Baseline	0.79 (0.13)	0.91 (0.13)
Post-Intervention 6-week follow-up	0.96 (0.13)*,****	0.78 (0.13)
Pre-Booster 22-week follow-up	1.10 (0.13)***,******	0.68 (0.13)*
Post-Booster 28-week follow-up	0.95 (0.13)*****	0.73 (0.13)
Refined grains		
Pre-Intervention Baseline	2.47 (0.17)	2.27 (0.17)
Post-Intervention 6-week follow-up	2.37 (0.17)	2.49 (0.17)
Pre-Booster 22-week follow-up	2.33 (0.17)*****	2.69 (0.17)**
Post-Booster 28-week follow-up	2.46 (0.17)	2.54 (0.17)
Meats/beans/eggs/nuts		
Pre-Intervention Baseline	1.07 (0.08)	1.17 (0.08)
Post-Intervention 6-week follow-up	1.13 (0.08)	1.13 (0.08)
Pre-Booster 22-week follow-up	1.14 (1.08)	1.09 (0.08)
Post-Booster 28-week follow-up	1.13 (1.08)	1.13 (0.08)
Dairy		
Pre-Intervention Baseline	0.73 (0.07)	0.76 (0.07)
Post-Intervention 6-week follow-up	0.79 (0.07)	0.73 (0.07)
Pre-Booster 22-week follow-up	0.73 (0.07)	0.80 (0.07)
Post-Booster 28-week follow-up	0.74 (0.07)****	0.63 (0.07)**

*within-groups change baseline to follow-up *P* < .05, **P < .01, ***P < .001

****treatment-by-time interaction baseline to follow-up *P* < .05, *****P < .01, ******P < .001

Means and standard errors are based on mixed-effects models of the treatment-by-time interaction that adjusted for repeated measures within families and nesting of families within ECE centers. The models were based on data from 4712 lunches packed by parents for their preschool children. Each model compared the given measurement period to baseline. The models also were adjusted for gender, age, heritage, parental marital status and parental education

Servings of whole grains that parents packed in the children's bag lunches at the intervention centers rose from 0.79 at baseline to 0.96 at the post-intervention 6-week follow-up and was maintained during the 3 months of non-intervention (1.10 servings at the pre-booster 22-week follow-up) and after the booster (0.95 servings at the post-booster 28-week follow-up). At the control centers the amount of whole grain items that parents packed in their children’s lunches was consistently less than one serving and did not differ from baseline. Consequently, the increase at the intervention centers relative to control centers was significant at the post-intervention 6-week follow-up (0.30 servings, SE = 0.13, *P* = 0.018), at the pre-booster 22-week follow-up (0.54 servings, SE = 0.13, *P* < 0.001), and at the post-booster 28-week follow-up (0.34 servings, SE = 0.13, *P* = 0.009). At none of the measurement periods did the amount of whole grains parents packed in their children’s lunches meet the “My Plate” recommendation to “make half your grains whole.” At the post-intervention 6-week follow-up and the post-booster 28-week follow-up, average servings of whole grains in the children’s lunches at the intervention centers was 0.96 and 0.95, respectively compared to the average of 2.37 and 2.46 servings of refined grains packed in the lunches.

There were no changes at intervention or control centers at any of the follow-up measurement periods in servings of meats/beans/eggs/nuts or of chips parents packed in their children’s bag lunches. Although the time-by-treatment interaction was significant for dairy at the 28-week post-booster follow-up (0.14 servings, SE = 0.05, *P* = 0.011), this effect was driven by decrease in servings of dairy at control centers rather than increase at intervention centers.

Servings of sweets (e.g., fruit drinks, cookies, candy) parents packed in the children’s lunches decreased from 1.25 servings at baseline to 1.15 servings at the pre-booster 22-week follow-up at the intervention centers. At the control centers, servings of sweets increased to 1.53 servings. These results are presented in Table 4. The difference in magnitude of change in intervention compared to control groups was significant (-0.43 servings, SE = 0.11, *P* < 0.001).

**Table 4:** Numbers of servings of chips and sweets observed in the children’s bag lunches

	Intervention Mean (Standard Error)	Control Mean (Standard Error)
Chips		
Pre-Intervention Baseline	0.41 (0.06)	0.54 (0.05)
Post-Intervention 6-week follow-up	0.37 (0.06)	0.50 (0.05)
Pre-Booster 22-week follow-up	0.38 (0.06)	0.47 (0.05)
Post-Booster 28-week follow-up	0.48 (0.06)*	0.51 (0.05)
Sweets		
Pre-Intervention Baseline	1.36 (0.13)	1.31 (0.12)
Post-Intervention 6-week follow-up	1.25 (0.13)	1.30 (0.12)
Pre-Booster 22-week follow-up	1.15 (0.13)**,******	1.53 (0.13)**
Post-Booster 28-week follow-up	1.34 (0.13)	1.46 (0.13)
TOTAL Chips & Sweets		
Pre-Intervention Baseline	1.92 (0.16)	2.00 (0.20)
Post-Intervention 6-week follow-up	1.80 (0.16)	1.90 (0.20)
Pre-Booster 22-week follow-up	1.75 (0.16)*	2.10 (0.20)
Post-Booster 28-week follow-up	1.99 (0.16)	2.10 (0.20)

*within-groups change baseline to follow-up *P* < .05, **P < .01, ***P < .001

****treatment-by-time interaction baseline to follow-up *P* < .05, *****P < .01, ******P < .001

Means and standard errors are based on mixed-effects models of the treatment-by-time interaction that adjusted for repeated measures within families and nesting of families within ECE centers. The models were based on data from 4712 lunches packed by parents for their preschool children. Each model compared the given measurement period to baseline. The models also were adjusted for gender, age, heritage, parental marital status and parental education. Percent of parents packing the given foods was based on the occurrence of any amount of the food on at least one of the two days in the measurement period

Irrespective of whether the observations were at intervention or at control centers, parents packed their children’s lunches with small amounts of vegetables (ranging from 0.24 to 0.52 servings on average at the different measurement periods) and large amounts of sweets and chips (ranging from 1.75 to 2.10 servings on average at the different measurement periods).

### Changes in prevalence of parents’ packing healthy choices in their children’s bag lunches

Percent of parents packing the specified foods on at least one of the two days in the given measurement period is presented in Table 5. At baseline at intervention and at control centers at each measurement period, fruit, grains, meats/beans/eggs/nuts, and dairy were packed by more than 86 % of parents at least occasionally whereas prevalence of packing whole grains was less than 52 % and, except in immediate response to intervention at the 6-week and 28-week follow-ups, prevalence of packing vegetables was less than 66 %.

**Table 5:** Percent of parents who packed foods from the “My Plate” groups in the child’s lunch

	Intervention Percent (Standard Error)	Control Percent (Standard Error)
Fruit		
Pre-Intervention Baseline	91.3 (2.6)	89.4 (2.5)
Post-Intervention 6-week follow-up	93.7 (2.6)****	86.3 (2.5)
Pre-Booster 22-week follow-up	93.2 (2.6)****	86.1 (2.6)
Post-Booster 28-week follow-up	92.6 (2.6)	87.7 (2.6)
Vegetables		
Pre-Intervention Baseline	52.8 (4.7)	45.8 (4.5)
Post-Intervention 6-week follow-up	70.9 (4.7)***,******	42.6 (4.6)
Pre-Booster 22-week follow-up	58.2 (4.7)	45.9 (4.6)
Post-Booster 28-week follow-up	65.1 (4.7)***,*****	45.5 (4.7)
Whole grains		
Pre-Intervention Baseline	41.9 (5.0)	45.7 (5.0)
Post-Intervention 6-week follow-up	47.3 (5.0)****	39.1 (5.0)
Pre-Booster 22-week follow-up	51.7 (5.1)**,******	36.9 (5.1)*
Post-Booster 28-week follow-up	46.8 (5.1)*****	36.0 (5.1)*
Refined grains		
Pre-Intervention Baseline	91.0 (3.3)	85.8 (3.2)
Post-Intervention 6-week follow-up	86.4 (3.3)	86.9 (3.3)
Pre-Booster 22-week follow-up	80.9 (3.3)***,*****	87.8 (3.3)
Post-Booster 28-week follow-up	86.7 (3.3)	85.2 (3.3)
Meats/beans/eggs/nuts		
Pre-Intervention Baseline	89.3 (2.9)	93.5 (2.9)
Post-Intervention 6-week follow-up	90.1 (2.9)	93.3 (2.9)
Pre-Booster 22-week follow-up	93.4 (3.0)	92.9 (3.0)
Post-Booster 28-week follow-up	87.6 (3.0)	91.9 (3.0)
Dairy		
Pre-Intervention Baseline	82.3 (3.2)	83.8 (3.1)
Post-Intervention 6-week follow-up	87.5 (3.2)	82.9 (3.2)
Pre-Booster 22-week follow-up	81.1 (3.2)	84.7 (3.2)
Post-Booster 28-week follow-up	84.4 (3.2)	82.4 (3.2)

*within-groups change baseline to follow-up *P* < .05, **P < .01, ***P < .001

****treatment-by-time interaction baseline to follow-up *P* < .05, *****P < .01, ******P < .001

Percentages and standard errors are based on mixed-effects models of the treatment-by-time interaction that adjusted for repeated measures within families and nesting of families within ECE centers. The models were based on data from 4712 lunches packed by parents for their preschool children. Each model compared the given measurement period to baseline. The models also were adjusted for gender, age, heritage, parental marital status and parental education. Percent of parents packing the given foods was based on the occurrence of any amount of the food on at least one of the two days in the measurement period

Time by treatment analysis showed intervention effects on prevalence of parents packing of fruit, vegetables, and whole grains. Effect size was computed as the difference in magnitude of change from baseline- to 6-week follow-up in the intervention group net of the change observed in the control group. Effect sizes were 5.5 % (SE = 2.4, *P* = 0.021) for fruit, 21.3 % (SE = 4.7, *P* < 0.001) for vegetables, and 12.1 % for whole grains (SE = 5.4, *P* = 0.027). Increased prevalence of packing was maintained at the pre-booster 22-week follow-up for fruit (5.1 %, SE = 2.4, *P* = 0.033) and for whole grains (18.6 %, SE = 5.5, *P* < 0.001), but not for vegetables. At the post-booster 28-week follow-up, increased prevalence of packing whole grains was maintained (14.6 %, SE = 5.5, *P* = 0.008), and increased prevalence of parents’ packing of vegetables in their children’s lunches was renewed (12.7 %, SE = 4.8, *P* = 0.008).

Information about prevalence of parents packing chips and sweets in their preschoolers’ bag lunches is presented in Table 6. On average, more than a third of parents packed chips in their children’s lunches on at least one of the two days observed in the measurement periods, and approximately two-thirds packed sweets. At each follow-up, prevalence of packing chips decreased from baseline at the control centers, and the difference in amount of change compared to the intervention centers was 12.8 % (SE = 5.0, *P* = 0.011). At the 6-week and 22-week follow-up observations, prevalence of packing sweets decreased from baseline at the intervention centers and was manifested as a significantly sharper decline from baseline to 22-week pre-booster follow-up compared to the control centers (-9.9 %, SE = 4.9, *P* = 0.044).

**Table 6:** Percent of parents who packed chips and sweets in the child’s lunch

	Intervention Percent (Standard Error)	Control Percent (Standard Error)
Chips		
Pre-Intervention Baseline	35.1 (4.5)	48.0 (4.4)
Post-Intervention 6-week follow-up	30.7 (4.5)	39.7 (4.4)*
Pre-Booster 22-week follow-up	35.0 (4.5)	40.6 (4.5)*
Post-Booster 28-week follow-up	40.1 (4.5)	40.2 (4.5)*,****
Sweets		
Pre-Intervention Baseline	68.5 (4.7)	66.4 (4.6)
Post-Intervention 6-week follow-up	61.9 (4.7)*	65.4 (4.6)
Pre-Booster 22-week follow-up	61.2 (4.7)*,****	69.0 (4.7)
Post-Booster 28-week follow-up	64.4 (4.8)	68.0 (4.7)

*within-groups change baseline to follow-up *P* < .05, **P < .01, ***P < .001

****treatment-by-time interaction baseline to follow-up *P* < .05, *****P < .01, ******P < .001

Means and standard errors are based on mixed-effects models of the treatment-by-time interaction that adjusted for repeated measures within families and nesting of families within ECE centers. The models were based on data from 4712 lunches packed by parents for their preschool children. Each model compared the given measurement period to baseline. The models also were adjusted for gender, age, heritage, parental marital status and parental education. Percent of parents packing the given foods was based on the occurrence of any amount of the food on at least one of the two days in the measurement period

### Changes in parents’ habit of packing healthy meal pattern in their preschool children’s bag lunches

Frequency of lunches qualifying for “gold medal” by containing all five of the “My Plate” food groups (fruit, vegetables, grains/whole grains, dairy, meats/beans/eggs/nuts) was less than five percent at baseline at intervention and at control centers. At intervention centers, parents’ frequency of packing lunches that qualified as “gold medal” increased to 11 % at the post-intervention 6-week follow-up and the time by treatment interaction was significant (proportion difference of 0.10, SE = 0.02, *P* < 0.001). The results presented in Table 7 show that the difference between intervention and control groups was maintained at the pre-booster 22 week follow-up (mean difference of 0.06, SE = 0.02, *P* = 0.008). There was, however, no evidence the booster week of *Lunch is in the Bag* effected any further “boost” in number of days on which parents packed bag lunches with all five of the My Plate food groups.

**Table 7:** Proportion of parent-packed lunches that presented healthy meal patterns

	Intervention Percent (Standard Error)	Control Percent (Standard Error)
Contained foods from all 5 of the My Plate food groups (Gold Medal)		
Pre-Intervention Baseline	0.03 (0.02)	0.04 (0.02)
Post-Intervention 6-week follow-up	0.11 (0.02)***,******	0.01 (0.02)
Pre-Booster 22-week follow-up	0.07 (0.02)**,*****	0.02 (0.02)
Post-Booster 28-week follow-up	0.07 (0.02)*	0.03 (0.02)
Contained no sweets or chips		
Pre-Intervention Baseline	0.36 (0.4)	0.34 (0.04)
Post-Intervention 6-week follow-up	0.42 (0.04)*	0.35 (0.04)
Pre-Booster 22-week follow-up	0.41 (0.04)*	0.33 (0.04)
Post-Booster 28-week follow-up	0.38 (0.04)	0.36 (0.04)

*within-groups change baseline to follow-up *P* < .05, **P < .01, ***P < .001

****treatment-by-time interaction baseline to follow-up *P* < .05, *****P < .01, ******P < .001

Proportions and standard errors are based on mixed-effects models of the treatment-by-time interaction that adjusted for repeated measures within families and nesting of families within ECE centers. The models were based on data from 4712 lunches packed by parents for their preschool children. Each model compared the given measurement period to baseline. Proportion of Gold Medal lunches was based on occurrence of all five food groups irrespective of serving size

At each measurement period in the intervention and in the control groups, less than half of the parent-provided bag lunches excluded sweets and chips. At the intervention centers there were indications of increases at 6-week and 22-week follow-up in parents’ frequency of packing lunches without sweets and chips, but the changes were too small to be statistically significant in comparison to the control centers. Even after the intervention, sweets (e.g., sugar-sweetened beverages, cookies, candy) together with high fat, high sodium chips persisted in accounting for a combined total of nearly two servings of food in the children’s lunches.

## Discussion

*Lunch is in the Bag* had some positive effects on parents’ lunch packing behaviors. It increased servings of whole grains, prompted temporary increase in servings of vegetables, prevented increase in servings of sweets parents packed in their preschool children’s bag lunches, and increased the percent of lunches containing items from all five of the “My Plate” food groups. Although there was no effect on servings of fruit, the intervention did increase the percent of parents who packed fruit in their children’s lunches at least occasionally and also increased prevalence of parents who packed vegetables and whole grains.

Packing fruit in the lunch for a 3 to 5 year old child to eat at the ECE center thus appeared to be normative behavior, but despite the intervention’s positive effects, packing vegetables and whole grains was not normative. Across measurement periods and treatment groups, nearly all parents (i.e., more than 86 %) packed fruit at least occasionally and the average amount packed was more than one full age-appropriate serving. In contrast, even after the intervention only about half of the parents packed whole grains and about two-thirds packed vegetables in their children’s lunches at least occasionally, and the average amount of vegetable packed persisted in being substantially less than a full age-appropriate serving.

These findings help to explain why, at intervention and control centers at each measurement period, less than 15 % of the children’s bag lunches contained items from all five of the “My Plate” food groups. Sweets and/or chips continued to be frequent items in the children’s parent-provided bag lunches. Even after the intervention, more than half of the lunches contained sweets (e.g., sweetened fruit drinks) and/or high fat/high sodium chips (e.g., cheese crackers), and the average amount of sweets and chips continued to exceed one age-appropriate serving.

*Lunch is in the Bag*’s efficacy trial supported the hypothesis that at the 6-week and 28-week follow-up, compared to children’s lunches at the control centers, children’s lunches in the intervention group would contain more servings of whole grains. The average amount of whole grains parents packed in their children’s lunches at the intervention centers increased to very near the intervention target of a full age-appropriate serving. The study results also provided some support for the hypothesis that the intervention would increase vegetables in the children’s lunches. The initial effect for vegetables was an increase from substantially less than half a serving to approximately half a serving. Although there was small positive response to the re-introduction of intervention messages during the week of booster activities, parents’ packing of vegetables in the intervention group did not overcome the degradation of effect that occurred during the three months of non-intervention. At the 28-week follow-up, the amount of change from baseline in servings of vegetables packed in children’s lunches at the intervention centers was not different from the uniformly small amount of vegetables packed by parents at the control centers.

These results confirmed speculative conclusions from prior work that behavior change pathways differ across food items [23, 39, 48]. Prevalence of parents packing vegetables in their children’s lunches increased responsive to the initial five weeks of intervention, then decreased in the absence of intervention activities, but rebounded when the booster week re-introduced the intervention messages, cues and reinforcements. In contrast, the prevalence of parents packing whole grains in their children’s lunches increased responsive to the initial five weeks of intervention and the increase was maintained—i.e., was not affected by the removal or subsequent re-introduction of the intervention messages, cues, and reinforcements.

These differences in response to intervention provide endorsement of food-specific approaches to increase the array of healthy options and decrease the persistent prevalence of less healthy options in young children's parent-packed bag lunches. Qualitative research indicates that parents are positively disposed to the sensory characteristics of whole-grain products but have only limited knowledge about ways to identify these foods [30]. An implication is that a cognitive-based intervention approach is especially appropriate for getting parents to pack more whole grains in their preschool children's bag lunches. It appears that, once parents know how to identify whole grain foods at time of purchase and see that their children will accept whole grain bread for sandwich or brown rice in a casserole-type dish, they are willing to regularly use the products. In addition, largely due to the Dietary Guidelines recommendations in 2005, whole grain products have become more prevalent in grocery stores in the USA and are available in many family products, and so are relatively easy and convenient to substitute for refined grain products. Thus, the food environment is likely a factor that influences response to interventions designed to increase packing of whole grain products.

Vegetables appear to be a more challenging objective for behavioral intervention. Laboratory study [49] and survey of preschool children's food preferences [50] show vegetables to be liked less compared to cereals and fruit. Because of concerns about the cost of food waste and/or desire to please the child, parents may be reluctant to pack their child's lunch bag with foods they believe the child dislikes or will not eat [32, 51]. *Lunch is in the Bag*'s classroom activities for children and center-based parent-child learning stations were intervention components with potential for getting parents and children to agree the child likes and willingly consumes vegetables. The Lunch Colors classroom activity included awarding a "gold medal" certificate to the parents when the child's lunch included foods from all five of the My Plate food groups. But neither the initial five weeks of intervention nor the addition of a booster week of intervention was sufficient to increase parents' packing of vegetables to a level comparable to the already normative practice of packing fruit in their children's lunch bags.

The up-down-up again prevalence of packing vegetables in response to the introduction-withdrawal-reintroduction of the *Lunch is in the Bag* intervention suggests additional attention should be directed toward intervention strategies that increase the amounts of social and emotional gratification parents receive from providing their children with vegetables [39]. The high level of challenge in this task is underscored in the baseline data showing that the participating parents expressed less intention to pack vegetables in their children’s lunches and were less endorsing of packing vegetables compared to fruit and whole grain items (e.g., “taste good,” “easy to pack,” “child likes them”). Given these circumstances, it is possible that children complain when their parents increase the amount of vegetables packed in the lunch. Other research suggests, however, that preschool aged children rarely talk to their parents about vegetables packed in their bag lunches [52]. As there are few options currently available in grocery stores in the USA for single serve packaged vegetables, the food environment likely is a factor that contributes to the challenge of increasing parents’ packing of vegetables in lunches for their young children to eat at the ECE center.

This current study provided minimal support for the hypothesis that increasing parents’ packing of fruit, vegetables, and whole grains would displace chips and sweets thereby increasing children’s exposure to healthy meal pattern in the lunch they bring from home. The intervention had the apparent effect of preventing increase in servings of sweets in the children’s lunches, but at each measurement time period in both the intervention and the control groups, the amount of sweets parents packed in their children’s lunches was, on average, more than one full age-appropriate serving. These results reinforce conclusions drawn by others [53, 54] that, in addition to providing parents with messages and activities to increase prevalence and strengthen the habit of choosing healthy foods for their preschoolers, parents also need messages and activities and reminders focused on what to omit from the lunches and how to resist normative and other pressures to pack foods that are high in added fat/salt/sugar in their children’s lunches.

Outcomes of the *Lunch is in the Bag* efficacy trial add to the accumulating evidence that parents need more and better assistance to leverage the opportunity a packed lunch provides for helping children to learn to eat and enjoy vegetables, whole grains and other healthy choices instead of foods high in added fat, sugar, and sodium such as chips and sweets. Although the *Lunch is in the Bag* newsletters sent from the ECE center to the parents provided sample menus and information focused on maximizing healthy options, observation of the children's lunches at baseline and at follow-up suggested parents packed to accommodate or please the child by packing ‘kid foods’ [55, 56] such as fruit leathers and similar sweetened fruit snacks. In focus group research, school age children identify sweets and chicken nuggets and products with child-oriented packaging as ‘kid foods’ while unprocessed fruit, vegetables, and meats are perceived as ‘adult foods’ [55].

The idea that parents pack the lunch to please the child rather than to inculcate healthy eating patterns is reinforced in other research conducted with the population of families that participated in the trial for *Lunch is in the Bag.* That research showed the families’ home food inventory assessed with a validated food checklist [57] included large variety of vegetables and relatively less availability of sweets and unhealthy salty snacks; but the lunches they packed for their preschool children seldom contained vegetables and often contained sweets and/or chips [58]. The disconnect between home food inventory and food in the children’s lunch bags suggests the possibility that the chips and sweets in the home inventory were purchased specifically for lunch packing, responsive perhaps to children’s requests and/or the parents’ expectations of what the child will be happy to find in the lunch bag. Children are estimated to influence one-third to one-half of family food purchases [59, 60], with preschool-aged children making more purchase requests than older children [61], and requests for sweets and snack foods accounting for the largest share of young children’s in-store food requests [62].

Additional research is needed to understand better the forces that influence parents’ decisions about food for their preschoolers so that barriers can be removed and/or levers discovered to improve parents’ use of the lunch bag as a tool for helping their children to achieve healthy eating patterns. Future work should include qualitative studies with parents and children to identify how family traditions and food marketing influence the contents of young children’s bag lunches as well as communications research on how to encourage healthy choices and at the same time discourage the packing of sweets and chips in young children’s lunches.

There also is need for additional research focused on leveraging the collaborative relationship providers of ECE have with families to support the development of healthy eating behaviors during the crucially important early childhood years. The alliance families have with their children’s ECE caregivers holds strong potential as a natural source of support for the child’s healthy eating [63]. Other research conducted with the population of families and ECE caregivers that participated in the trial for *Lunch is in the Bag* showed parents perceived very little social support from their child’s ECE caregivers about the child’s eating behaviors [52], and the majority of the ECE caregivers indicated they seldom or never talk with parents about healthiness of the child’s eating behavior [2]. In the current study of *Lunch is in the Bag*, newsletters to parents was the component that most often obtained high implementation scores while center-level advocacy of parent and child nutrition education and behavior was the component that most often obtained low implementation scores. These circumstances perhaps explain why the primary outcomes did not reflect substantial improvement over those obtained in the pilot studies. The pilot version of *Lunch is in the Bag* increased servings of vegetables from 0.41 at baseline to 0.65 at the 6-week follow-up [1]; and in the study conducted to pilot test the addition of a booster week of activities, the obtained pattern for vegetables was 0.35 servings at baseline, increased to 0.50 at the 6-week follow-up, degraded to 0.35 at the 22-week follow-up, and rebounded to 0.45 at the 28-week follow-up [39]. These accumulated results emphasize need for additional research focused on discovery of effective means for strengthening the alliance of parents and ECE caregivers working together to support the establishment of healthy eating behaviors in early childhood. Such options could include research on policy approaches for assisting ECE centers as nutrition advocates and educators as well as approaches for improving implementation support for evidence-based nutrition behavior change interventions designed for delivery via the ECE center.

A limitation of the current study that also characterized the pilot studies was the over representation of higher educated, higher income, English-speaking families compared to the general population of families with young children residing in central Texas. This situation may be due to higher rates of participation by lower income families in subsidized ECE centers that serve hot meals reimbursed through the CACFP. None of the families who participated in the trial of Lunch is in the Bag requested Spanish translations of the intervention materials, but the absence of dual language versions of the newsletters may have limited their effectiveness and/or their attractiveness for recruiting participation of ECE centers and families. Although the sample of adults enrolled in the study was higher income and better educated than expected, assessment of the children’s weight status showed 22 % with overweight or obesity, and 6 % were underweight. These results indicate need for nutrition behavior change interventions targeted to families of all levels of income and education.

A notable strength of the study was the high rate at which ECE centers and participating families were retained. This result not only lends confidence in interpreting the outcomes but also reinforces conclusions reached in the formative phases of *Lunch is in the Bag*’s development [1, 33] that interventions that leverage the ECE center as a platform for assisting home-based changes to develop healthy eating patterns in early childhood are wanted by parents and by ECE personnel.

## Conclusions

This study demonstrated the need for and positive effects of implementing the *Lunch is in the Bag* intervention at ECE centers where parents send bag lunch for their young children. The intervention increased the numbers of servings of vegetables (increase from less than half a serving to approximately half a serving) and of whole grains (increase from slightly less than a full-serving to approximately a full serving). These positive changes in parents’ lunch packing behaviors did not, however, displace their packing of sweets and chips. Before and after the intervention, parents very rarely packed their children’s lunches with foods from all five of vegetables, fruit, grains, dairy, meats/beans/eggs/nuts, but often included one or more servings of sweets and chips. An important direction for future research is discovery of options for further strengthening and refining the intervention messages targeted to parents and their preschool aged children—e.g., identifying strategies to assist parents in resisting pressures to pack foods that are high in added fat, salt, and sugar such as chips and sweets. Another important direction for the future is the development of more options for leveraging the partnership of ECE centers and families to improve use of the bag lunch as a tool for assisting children to learn to eat and enjoy vegetables and other healthy foods in preference to less healthy choices such as chips and sweets.

## Abbreviations

ECE: Early Care and Education
BMI: Body Mass Index
